# A Cohort Study of the Age at Menopause in Female Patients With and Without Inflammatory Bowel Disease

**DOI:** 10.1093/crocol/otac027

**Published:** 2022-08-04

**Authors:** Varun P Moktan, Nader D Daoud, William J Tremaine, Edward V Loftus, Sunanda V Kane, Alexander P Hochwald, David O Hodge, Jana G Hashash, Stephanie S Faubion, Francis A Farraye

**Affiliations:** Division of Community Internal Medicine, Mayo Clinic, Jacksonville, Florida, USA; Division of Gastroenterology and Hepatology, Mayo Clinic, Jacksonville, Florida, USA; Division of Gastroenterology and Hepatology, Mayo Clinic, Rochester, Minnesota, USA; Division of Gastroenterology and Hepatology, Mayo Clinic, Rochester, Minnesota, USA; Division of Gastroenterology and Hepatology, Mayo Clinic, Rochester, Minnesota, USA; Department of Biomedical Statistics and Informatics, Mayo Clinic, Jacksonville, Florida, USA; Department of Biomedical Statistics and Informatics, Mayo Clinic, Jacksonville, Florida, USA; Division of Gastroenterology and Hepatology, Mayo Clinic, Jacksonville, Florida, USA; Division of General Internal Medicine, Mayo Clinic, Jacksonville, Florida, USA; Division of Gastroenterology and Hepatology, Mayo Clinic, Jacksonville, Florida, USA

**Keywords:** IBD, menopause, sex

## Abstract

**Background:**

Menopause, defined by the complete cessation of menstrual cycles for 12 consecutive months, may occur at a younger age in women who have concomitant immune dysregulation. Our aim was to determine whether women with inflammatory bowel disease (IBD) experience an earlier onset of menopause compared to women without IBD.

**Methods:**

This was a retrospective cohort study using resources of the Rochester Epidemiology Project, a collaboration between clinics, hospitals, and medical facilities in Olmsted County, Minnesota. From these people, women who were diagnosed with IBD between 1970 and 2010 comprised the case cohort while the reference cohort included women with no diagnosis of IBD. Data including age, body mass index (BMI), ethnicity, smoking status, age at onset of menopause, and current use of hormone therapy were collected. Patients with history of hysterectomy or oophorectomy were excluded. Wilcoxon rank-sum test for numeric variables and Fisher’s exact test for categorical variables were used to analyze the data.

**Results:**

A total of 171 women met criteria for inclusion (83 cases and 88 controls). Mean age of menopause in women with IBD was 50.0 years compared to 51.5 years in women with no IBD (*P* = .006). There was no difference in BMI of women with and without IBD (28.7 versus 28.2 kg m^−2^; *P* = .9), respectively. There were more former smokers (33.7%) and current (6%) smokers in the IBD group (*P* = .009) compared to the non-IBD group.

**Conclusions:**

IBD is associated with an earlier onset of menopause. Although it is unclear if this mean difference of 1.5 years is clinically relevant, it is known that early menopause is associated with an increased risk of osteoporosis and cardiovascular disease. Further research on the possible mechanisms is needed.

## Introduction

Menopause is defined as the absence of menstrual cycles for 12 consecutive months. In the United States, the average age of menopause is 51 years. There is a subset of women who undergo menopause at an earlier age due to underlying genetic, infectious, iatrogenic, autoimmune, or idiopathic causes. In 1 study of 48 women, a possible association between inflammatory bowel disease (IBD) and earlier age at menopause was seen.^1^ In that study, the authors found that women with Crohn’s disease had a mean age at menopause of 47.6 years compared to 49.6 years in a cohort of healthy women. In another study involving 65 women (20 with ulcerative colitis and 45 with Crohn’s disease), this associated was not confirmed.^2^ One abstract in 2011 showed that women with IBD had an almost 4-fold risk for developing premature ovarian insufficiency.^3^ The authors hypothesized that various chronic inflammatory conditions including IBD require continual immunosuppression which may accelerate reproductive senescence. Our aim was to determine whether women with IBD experience an earlier onset of menopause compared to women without IBD.

## Methods

The Rochester Epidemiology Project (REP), a shared diagnostic index that includes medical information on a population of patients residing in Olmsted County, Minnesota was used to conduct this study. We performed a historical cohort study to identify a group of county female residents who were diagnosed with IBD between 1970 and 2010 and respective controls who did not have IBD.

Demographics and clinical variables that included diagnosis of IBD, age, body mass index (BMI), race/ethnicity, smoking status, age at menopause, and any use of hormone therapy were collected.

Summary statistics were in the form of percentages for categorical variables and means with SDs for continuous variables of normal distribution. Medians and interquartile ranges were reported for non-normal data. The study started with the group of patients with IBD from the REP database, and a matched reference group of women who had menopause but did not have IBD. Overall comparisons of baseline factors were performed between those groups using Fisher’s exact test for categorical variables. Continuous variables were compared with a Wilcoxon rank-sum test. Analysis was completed using R-studio version 4.0.3 software. An adjusted *P* value of less than .05 was considered significant.

The average age of menopause in the United States is 51 years. For the purpose of power calculations, we hypothesized an average age of menopause in women with IBD at 47 years. At a power of greater than 90% to confirm this difference, we would need 100 patients within each group. Thus, we initially aimed for a sample size of 200 patients.

## Results

A total of 171 women met criteria for inclusion, 83 had IBD and 88 served as controls. In our cohort, 53% of the women had ulcerative colitis and 47% Crohn’s disease. Patient demographics, primary and secondary outcomes are shown in Table 1. The average age at onset of menopause in women with IBD was 50.0 years compared to 51.5 years in women without IBD (*P* = .006). The mean menopause age for Crohn’s patient was 50.5 years and the mean age for ulcerative colitis patients was 49.3 years. There were more former smokers (33.7%) and current (6%) smokers in the IBD group (*P* = .009) compared to the non-IBD group. Additionally, there were no differences in history of smoking between IBD subtypes (Table 2). There was no difference in BMI, race, and use of hormone therapy between the 2 groups.

**Table 1.** Patient demographics, primary and secondary outcomes.

Variable	Case patients (*N* = 83)		Control patients (*N* = 88)		*P*
	*N*	Median (minimum, maximum) or No. (%) of patients	*N*	Median (minimum, maximum) or No. (%) of patients	
Age at menopause (years)	83	50.0 (42.0, 61.0)	88	51.5 (37.0, 59.0)	.006
BMI	83	26.4 (18.7, 50.0)	88	27.2 (17.8, 47.8)	.899
IBD type	83		0		N/A
Crohn’s disease		39 (47.0%)		0 (0%)	
Ulcerative colitis		44 (53.0%)		0 (0%)	
Race	83		88		.338
White		81 (97.6%)		83 (94.3%)	
Black		0 (0.0%)		3 (3.4%)	
Asian		2 (2.4%)		2 (2.3%)	
Smoking	83		88		.009
Never smoked		50 (60.2%)		71 (80.7%)	
Former smoker		28 (33.7%)		13 (14.8%)	
Current smoker		5 (6.0%)		4 (4.5%)	
Hormone therapy (No.)	83	46 (55.4%)	88	50 (56.8%)	.878

Abbreviations: BMI, body mass index; IBD, inflammatory bowel disease.

**Table 2.** Smoking status between IBD subtypes.

Variable	Crohn’s patients (*N* = 39)		UC patients (*N* = 44)		*P*
	*N*	Median (minimum, maximum) or No. (%) of patients	*N*	Median (minimum, maximum) or No. (%) of patients	
Smoking	39		44		.165
Never smoked		25 (64.1%)		25 (56.8%)	
Former smoker		10 (25.6%)		18 (40.9%)	
Current smoker		4 (10.3%)		1 (2.3%)	

Abbreviations: IBD, inflammatory bowel disease; UC, ulcerative colitis.

## Discussion

Our study has shown that women with IBD have an earlier onset of menopause when compared to women with no IBD. The mean age of menopause in women with IBD was 50.0 versus 51.5 years in women without IBD (*P* = .006). Our data does point to the possibility that having a chronic inflammatory condition may accelerate the normal decline in ovarian function associated with menopause. IBD is an autoimmune disorder that often requires immunosuppressive therapies.^4^ Other studies show that women with autoimmune disorders such as type 1 diabetes mellitus (DM), rheumatoid arthritis, or systemic lupus erythematosus (SLE) also have an earlier onset of menopause. In a prospective study, women who developed type 1 DM before menarche were found to have delayed menarche and earlier age of menopause.^5^ Similarly, in a cross-sectional study of women with SLE, age of natural menopause was significantly younger among patient with SLE (47 years) when compared with healthy individuals (50.5 years).^6^ We contend that IBD has similar immune mechanisms that lead to chronic inflammation and earlier onset of menopause.

A major strength of our study was using the REP as it has been used and validated in seminal publications on population health. Furthermore, with the REP, we have been able to access scanned paper charts of patients from the 1940s and 1950s and have been able to add them our cohort, allowing for a better representation of the population. Additionally, our study is only one of a few that address the association between IBD and menopause.

One weakness in this study was the definition of menopause. The Stages of Reproductive Aging Workshop criteria define menopause as the absence of menses for a year (in the absence of endometrial ablation, surgery, or hormonal manipulation).^7^ It is unclear if providers specifically followed these rigorous criteria since the diagnosis of menopause was obtained from notes on record review. Only a few providers used ICD9 or ICD10 codes for menopause. Given that these coding systems began in 1975 and 1999, respectively, it is possible that the diagnosis of menopause might have been missed prior to 1975. Another limitation is the lack of thorough medication histories that would allow us to determine the effect of immunosuppressive agents on the timing of menopause onset. In addition, women in the REP are overwhelmingly Caucasian and these findings may not apply to women of other races. Although our patients were predominantly Caucasian (97%), the REP has been used in previous large-scale studies and have been found to be representative of the US population as a whole. Other causes such as HIV/AIDS, genetic syndromes, and previous history of chemotherapy or radiation were not identified during our chart reviews. Finally, our study did not collect data between IBD and non-IBD patients on future or past gynecologic or hormonally related cancers (eg, uterine, breast, etc). However, this would also be an interesting in the future for further consideration. In this study, we demonstrated that women with IBD underwent menopause 1.5 years earlier than the control cohort. What are the implications of early menopause in women with IBD? Early menopause (before age 45 years) and premature menopause (before age 40 years) are associated with numerous potential adverse long-term consequences including increased risk of osteoporosis and premature cardiovascular disease. As subsets of women with IBD already have an increased risk of osteoporosis as well as cardiovascular disease, early menopause can further increase this risk. It is incumbent that providers who care for women with IBD are aware that early menopause is not benign and that these women be referred to discuss hormone replacement with their gynecologist or primary care provider.^8^

## Conclusion

In summary, we showed a possible link between IBD and an earlier onset of menopause. The relationship of age at menopause with autoimmune conditions, including IBD, is a topic that needs further investigation. Women with early or premature menopause should be referred to their gynecologist or primary care provider to discuss the risks and benefits of hormone replacement therapy.

## Data Availability

The data that support the findings of this study are available from the corresponding author upon reasonable request.
